# Ramucirumab‐containing chemotherapy for patients with gastrointestinal neuroendocrine carcinoma refractory/intolerant to platinum‐based chemotherapy: A multicenter observational retrospective study (WJOG13420G)

**DOI:** 10.1002/ijc.70053

**Published:** 2025-07-23

**Authors:** Yuki Matsubara, Toshiki Masuishi, Waki Hosoda, Hidekazu Hirano, Saori Mishima, Hiroyuki Takahashi, Tomoyuki Otsuka, Kenta Kawasaki, Takeshi Kawakami, Kazuhiro Yanagihara, Takaya Shimura, Masato Komoda, Kozue Murayama, Keiko Minashi, Yoshiyuki Yamamoto, Yudai Shinohara, Shinichi Nishina, Nobuyuki Musha, Kyoko Kato, Kentaro Kawakami, Katsunori Shinozaki, Kenji Tsuchihashi, Takayuki Ando, Yosuke Kito, Akitaka Makiyama, Seiichiro Mitani, Kaori Hino, Naoki Izawa, Isao Oze, Kei Muro

**Affiliations:** ^1^ Department of Clinical Oncology Aichi Cancer Center Hospital Nagoya Aichi Prefecture Japan; ^2^ Department of Gastroenterology and Gastrointestinal Oncology National Cancer Center Hospital East Kashiwa Chiba Prefecture Japan; ^3^ Department of Pathology and Molecular Diagnostics Aichi Cancer Center Hospital Nagoya Aichi Prefecture Japan; ^4^ Department of Gastrointestinal Medical Oncology National Cancer Center Hospital Tokyo Japan; ^5^ Department of Hematology and Medical Oncology Kanagawa Cancer Center Yokohama Kanagawa Prefecture Japan; ^6^ Department of Medical Oncology, Osaka International Cancer Institute Osaka City Osaka Prefecture Japan; ^7^ Division of Gastroenterology and Hepatology, Department of Internal Medicine Keio University School of Medicine Tokyo Japan; ^8^ Division of Gastrointestinal Oncology Shizuoka Cancer Center Nagaizumi Shizuoka Prefecture Japan; ^9^ Department of Medical Oncology Kansai Electric Power Hospital Osaka City Osaka Prefecture Japan; ^10^ Department of Gastroenterology and Metabolism Nagoya City University Graduate School of Medical Sciences Nagoya City Aichi Prefecture Japan; ^11^ Department of Gastrointestinal and Medical Oncology NHO Kyushu Cancer Center Fukuoka City Fukuoka Prefecture Japan; ^12^ Department of Gastroenterology Saitama Cancer Center Kitaadachi‐gun Saitama Prefecture Japan; ^13^ Clinical Trial Promotion Department Chiba Cancer Center Chiba City Chiba Prefecture Japan; ^14^ Department of Gastroenterology Faculty of Medicine, University of Tsukuba Tsukuba City Ibaraki Prefecture Japan; ^15^ Department of Hematology/Oncology Japan Community Healthcare Organization Kyushu Hospital Fukuoka City Fukuoka Prefecture Japan; ^16^ Department of Medical Oncology Kurashiki Central Hospital Kurashiki City Okayama Prefecture Japan; ^17^ Department of Surgery Saiseikai Niigata Hospital Niigata City Niigata Prefecture Japan; ^18^ Department of Medical Oncology National Hospital Organization Nagoya Medical Center Nagoya City Aichi Prefecture Japan; ^19^ Department of Medical Oncology Keiyukai Sapporo Hospital Sapporo City Hokkaido Prefecture Japan; ^20^ Division of Clinical Oncology Hiroshima Prefectural Hospital Hiroshima City Hiroshima Prefecture Japan; ^21^ Department of Medicine and Biosystemic Science, Graduate School of Medical Sciences Kyushu University Hospital Fukuoka City Fukuoka Prefecture Japan; ^22^ Department of Gastroenterology and Hematology Faculty of Medicine, Toyama University Hospital Toyama City Toyama Prefecture Japan; ^23^ Department of Medical Oncology Ishikawa Prefectural Central Hospital Kanazawa City Ishikawa Prefecture Japan; ^24^ Cancer Center Gifu University Hospital Gifu City Gifu Prefecture Japan; ^25^ Department of Medical Oncology, Faculty of Medicine Kindai University Osaka‐sayama City Osaka Prefecture Japan; ^26^ Department of Gastrointestinal Medical Oncology NHO Shikoku Cancer Center Matsuyama City Ehime Prefecture Japan; ^27^ Department of Clinical Oncology St. Marianna University School of Medicine Kawasaki City Kanagawa Prefecture Japan; ^28^ Division of Cancer Information and Control Aichi Cancer Center Research Institute Nagoya Aichi Prefecture Japan

**Keywords:** neuroendocrine carcinoma, platinum failure, progression‐free survival, Ramucirumab

## Abstract

No standard second‐line chemotherapy has been established for gastrointestinal neuroendocrine carcinoma (NEC). This study aimed to determine whether ramucirumab (RAM) is a treatment candidate in this setting. We retrospectively collected data from patients with gastric and colorectal NEC who received second‐line chemotherapy following platinum‐based chemotherapy. The pathological diagnosis of NEC was confirmed centrally according to the World Health Organization 2019 classification. We compared the clinical outcomes between second‐ or later‐line chemotherapy in the RAM group and second‐line chemotherapy in the non‐RAM group. One‐hundred patients diagnosed with NEC by central pathological review were studied. The RAM and non‐RAM groups included 44 (gastric/colorectal cancer, 34/10) and 56 (37/19) patients, respectively. In the RAM group, 68% of patients received RAM as second‐line chemotherapy. RAM was most frequently combined with weekly paclitaxel for gastric NEC and FOLFIRI for colorectal NEC. The RAM group showed better trends in overall survival and progression‐free survival than the non‐RAM group, with a median of 9.0 versus 5.6 months and 4.3 versus 1.8 months, respectively (hazard ratios [HR]: 0.75 [95% CI: 0.45–1.28] and 0.45 [0.27–0.75]). Efficacy was more pronounced in gastric NEC (PFS HR: 0.32, OS HR: 0.56) compared to colorectal NEC (PFS HR: 0.82, OS HR: 1.47). The objective response rate was significantly higher in the RAM group (44%) than in the non‐RAM group (6%), with notably higher responses observed in patients receiving paclitaxel plus RAM (62%). This study suggested that RAM‐containing chemotherapy, especially paclitaxel plus RAM for gastric NEC, seems promising and should be further investigated.

## What's New?

Platinum‐based chemotherapy is the first‐line treatment for unresectable gastrointestinal neuroendocrine carcinoma (NEC). Which treatments are most effective following platinum‐based regimens remains unclear. In this study, the authors compared clinical outcomes between NEC patients who received second‐ or later‐line treatments either with or without the angiogenesis inhibitor ramucirumab. Analyses show that ramucirumab often was combined with paclitaxel for gastric NEC or FOLFIRI for colorectal NEC, resulting in improved efficacy. Compared to non‐ramucirumab treatments, ramucirumab‐containing chemotherapy was associated with better survival. The findings indicate that second‐line ramucirumab‐containing regimens are highly effective in gastrointestinal NEC patients who are refractory to platinum‐based first‐line chemotherapy.

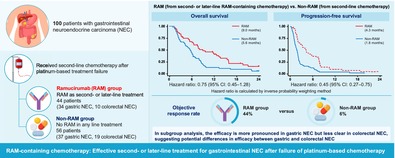

**Abbreviations**
CI: confidence interval
DCR: disease control rate
ECOG PS: Eastern Cooperative Oncology Group Performance Status
FTD/TPI: trifluridine/tipiracil
GI‐NEC: gastrointestinal neuroendocrine carcinoma
HR: hazard ratio
IPW: inverse probability weighting
NEC: neuroendocrine carcinoma
NEN: neuroendocrine neoplasm
ORR: objective response rate
OS: overall survival
PFS: progression‐free survival
RAM: ramucirumab
VEGF: vascular endothelial growth factor
VEGFR‐2: vascular endothelial growth factor receptor 2
WHO: World Health Organization
WJOG: West Japan Oncology Group

## 1 INTRODUCTION

Gastroenteropancreatic neuroendocrine neoplasms (NENs) are rare tumors, with neuroendocrine carcinoma (NEC) comprising only 6.2% of all NEN cases.^1^, ^2^ Advanced gastrointestinal neuroendocrine carcinoma (GI‐NEC) has a poor prognosis, with a median overall survival (OS) of 7.5–11.0 months.^3^, ^4^, ^5^ Despite limited clinical trials, platinum‐based treatment is recommended as the first‐line treatment for unresectable GI‐NEC^3^, ^6^, ^7^, ^8^, ^9^; however, no consensus has been reached on the second‐line treatment strategy, resulting in the adoption of various treatments according to the guidelines for gastrointestinal adenocarcinoma or small‐cell lung cancer.^10^ Previous reports, containing small cohorts, indicate limited efficacy of second‐line treatments such as FOLFIRI plus bevacizumab, liposomal irinotecan/5‐FU/folinic acid, docetaxel, FOLFOX, topotecan, and temozolomide‐based chemotherapy with an objective response rate (ORR) of 0–33%, progression‐free survival (PFS) of 2.0–6.0 months, and an OS of 3.2–22.0 months.^11^, ^12^, ^13^, ^14^, ^15^ These findings indicate an unmet need for an effective second‐line treatment for patients with GI‐NEC who are refractory or intolerant to first‐line platinum‐based chemotherapy.

Angiogenesis is associated with tumorigenesis and metastasis, during which vascular endothelial growth factor (VEGF) plays a central role. Therefore, VEGF inhibitors show an antitumor effect against various malignancies. Ramucirumab (RAM) is a fully human anti‐vascular endothelial growth factor receptor‐2 (VEGFR‐2) monoclonal IgG1 antibody, which exerts its anti‐angiogenic effect by specifically binding VEGFR‐2 and thereby blocking the binding of its ligands, VEGF‐A, VEGF‐C, and VEGF‐D.^16^ Chemotherapy with RAM is one of the standard treatments for gastric and colorectal adenocarcinoma^17^, ^18^, ^19^ Recent reports have suggested RAM plus chemotherapy as a potential treatment for gastric NEC^20^ Additionally, the use of VEGF inhibitors was shown to exert antitumor effects in a colon NEC xenograft model^21^ Since these reports contain a small number of patients, we investigated the efficacy and safety of the RAM‐containing chemotherapy for NEC, adding colorectal NEC in this multicenter study. We hypothesized that RAM could be administered based on the standard treatment for gastric and colorectal adenocarcinoma in daily practice.

## 2 MATERIALS AND METHODS

### 2.1 Patients

In this multicenter observational retrospective study, we examined patients with gastric or colorectal NEC previously treated with first‐line platinum‐based chemotherapy followed by second‐line chemotherapy between March 2015 and June 2020 (gastric NEC) or between May 2016 and June 2020 (colorectal NEC) at 26 facilities belonging to the West Japan Oncology Group (WJOG). These periods commence, respectively, with the approval of RAM in Japan for gastric and colorectal cancers. The patients were divided into two groups: those who received RAM‐containing chemotherapy as second‐line treatment or beyond (the RAM group) and those who did not receive RAM‐containing chemotherapy as any‐line treatment (the non‐RAM group) (Figure S1).

The inclusion criteria were as follows: age ≥20 years; histological diagnosis of NEC or mixed adenoneuroendocrine carcinoma based on the World Health Organization (WHO) 2010 classification of tumors of the digestive system; primary organ was the stomach or colorectum; unresectable distant metastasis or recurrent lesions; refractory or intolerant to platinum‐based chemotherapy; and Eastern Cooperative Oncology Group Performance Status (ECOG PS) 0–2 at the start of RAM‐containing chemotherapy (the RAM group) and at the start of second‐line treatment (the non‐RAM group). Patients who had previous administrations of RAM were excluded from this study.

### 2.2 Histological diagnosis and central pathology review

Most patients enrolled in this study were diagnosed at each participating institution according to the 2010 WHO classification. We performed a central pathological review to confirm the diagnosis of NEC using histological examination and immunolabelling. Central pathological evaluation was conducted by a pathologist with an expertise in neuroendocrine tumor pathology (WH). Hematoxylin and eosin staining and immunohistochemical staining of synaptophysin, chromogranin A, and Ki‐67/MIB‐1 were reviewed. If unstained tissue specimens were available, we added adjunct immunohistochemical markers (INSM‐1, Rb, and p53) for the differential diagnosis of NEC, as described previously.^23^ All patients were reclassified according to the 2019 WHO classification of tumors of the digestive system. In this study, patients diagnosed via biopsy were categorized into NEC and MANEC, and we categorized patients into MANEC only if a substantial amount of tumor cells showing NEC morphology and immunohistochemical findings (in at least 30% of tumor cells) were identified in their specimens. Pathological re‐evaluation was performed without providing any information on the treatment and outcome of each patient.

### 2.3 Outcomes and statistical analyses

The primary endpoint of the study was OS. The secondary endpoints included PFS, ORR, disease control rate (DCR), and adverse events associated with chemotherapy containing RAM. We evaluated the efficacy of second‐ or later‐line chemotherapy containing RAM (RAM group) compared with that of second‐line chemotherapy without RAM (non‐RAM group). In the RAM group, OS and PFS were defined from the initiation of RAM‐containing chemotherapy to the date of death due to any cause and to the date of disease progression or death, respectively, whichever occurred first. In the non‐RAM group, OS and PFS were defined similarly but were measured from the start of second‐line treatment. The best treatment responses were evaluated according to the Response Evaluation Criteria in Solid Tumors, version 1.1. The depth of response was defined as the rate of tumor shrinkage from that observed on the baseline computed tomography. Tumor responses were determined by the local investigators. Adverse events were assessed during RAM‐containing chemotherapy according to the Common Terminology Criteria for Adverse Events, version 5.0. In this study, we did not collect data on adverse events of grade 1 or 2 because these can be ambiguous and subject to different interpretations by each researcher, particularly in a retrospective analysis.

The median OS and PFS were calculated using the Kaplan–Meier method, and the treatment effect was evaluated using the hazard ratio (HR) and 95% confidence interval (CI) using the Cox proportional hazard model. The propensity score for receiving RAM‐containing chemotherapy was calculated using logistic regression with potential confounders as covariates. The inverse probability weighting (IPW) approach of the propensity score was used to consider potential bias.^22^ Age, sex, number of metastatic sites, stage, histology, ECOG PS, response to platinum therapy, number of prior regimens, previous primary resection, liver metastasis, lung metastasis, and peritoneal metastasis were used as covariates for both the IPW approach and multivariate analysis. The HR derived from the univariate analysis was defined as the crude HR, whereas the HR obtained from the multivariate analysis was referred to as the adjusted HR. Additionally, the HR analyzed using IPW was termed the PS‐IPW HR. Fisher's exact test and the Mann–Whitney *U* test were used to compare patient characteristics, ORR, and DCR between the groups. All statistical analyses were performed using STATA version 17.0, except for Sankey plots and Fisher's exact tests to compare patient characteristics, ORR, and DCR, which were performed using R version 4.3.1.

## 3 RESULTS

### 3.1 Patients

Initially, this retrospective study enrolled 165 patients with gastric or colorectal NEC from 26 facilities belonging to WJOG. Among these, 125 patients had archival histologic slides and/or unstained specimens available, which underwent a central pathology review for the confirmation of NEC diagnosis and reclassification based on the WHO 2019 classification of tumors of the digestive system, resulting in 100 patients being reclassified as having gastric or colorectal NEC and 25 cases being excluded (Figure S2). The reasons for exclusion were as follows: Ki‐67 not evaluated (*n* = 13), reclassification into non‐NEC tumors (poorly differentiated adenocarcinoma [*n* = 7], neuroendocrine tumor grade 2 [*n* = 2], and undifferentiated carcinoma not otherwise specified [*n* = 1]), and inadequate quality of tissue samples for diagnosis (*n* = 2). Among 100 patients diagnosed with NEC, 30 were administered RAM‐containing chemotherapy as second‐line therapy, nine as third‐line therapy, and five as fourth‐line therapy or beyond. Consequently, 44 and 56 patients were included in the RAM and non‐RAM groups, respectively (Figure S3). Tables 1 and S1 show the patient characteristics. The median Ki‐67 index was 80% (range 30–100) in the RAM group and 80% (range 40–100) in the non‐RAM group. Notably, only three patients in the RAM group and only one in the non‐RAM group had a Ki‐67 index of <50%. The non‐RAM group included more patients with synchronous metastatic disease and fewer previous platinum responders. On the other hand, there were more patients with liver or lung metastasis in the non‐RAM group. Additionally, eight patients with mixed adenoneuroendocrine carcinoma were observed in the RAM group, and four were observed in the non‐RAM group. In gastric NEC, the prevalence of patients with synchronous metastasis was higher in the non‐RAM group, while patients with liver metastasis were more frequent in the non‐RAM group. For colorectal NEC, there were no differences between the groups except in the number of prior treatment lines, which was an expected outcome based on the inclusion criteria. The efficacy of the first‐line treatment is summarized in Table S2 and Figure S4.

**TABLE 1:** Characteristics of patients.

Factor	RAM group *N* = 44	Non‐RAM group *N* = 56	*P* value
Age (years)			
Median (range)	70 (46–83)	68 (41–81)	0.27
Sex			
Male	36 (82%)	41 (73%)	0.35
Female	8 (18%)	15 (27%)	
Histology			
NEC	36 (82%)	52 (93%)	0.12
MANEC	8 (18%)	4 (7%)	
Time of first metastasis			
Synchronous	28 (64%)	46 (82%)	0.04
Metachronous	16 (36%)	10 18%	
Ki‐67			
Median (range)	80 (30–100)	80 (40–100)	0.96
≥50%	41 (93%)	55 (98%)	0.32
<50%	3 (7%)	1 (2%)	
ECOG PS			
0	20 (45%)	22 (39%)	0.82
1	21 (48%)	29 (52%)	
2	3 (7%)	5 (9%)	
Number of prior regimens			
1	30 (68%)	56 (100%)	<0.001
2	9 (20%)	0	
≥3	5 (11%)	0	
Response to platinum			
Yes	23 (68%)	14 (25%)	0.006
No	21 (32%)	42 (75%)	
Previous primary resection			
Yes	18 (41%)	16 (29%)	0.21
No	26 (59%)	40 (71%)	
Number of metastatic lesions			
1	20 (45%)	18 (32%)	0.21
≥2	24 (55%)	38 (68%)	
Liver metastasis			
Yes	33 (75%)	51 (91%)	0.05
No	11 (25%)	5 (9%)	
Lung metastasis			
Yes	2 (5%)	11 (20%)	0.04
No	42 (95%)	45 (80%)	
Peritoneal metastasis			
Yes	10 (23%)	11 (20%)	0.80
No	34 (77%)	45 (80%)	

Abbreviations: ECOG PS, Eastern Cooperative Oncology Group Performance Status; MANEC, mixed adenoneuroendocrine carcinoma; NEC, neuroendocrine carcinoma; RAM, ramucirumab.

### 3.2 Treatments

**FIGURE 1:**
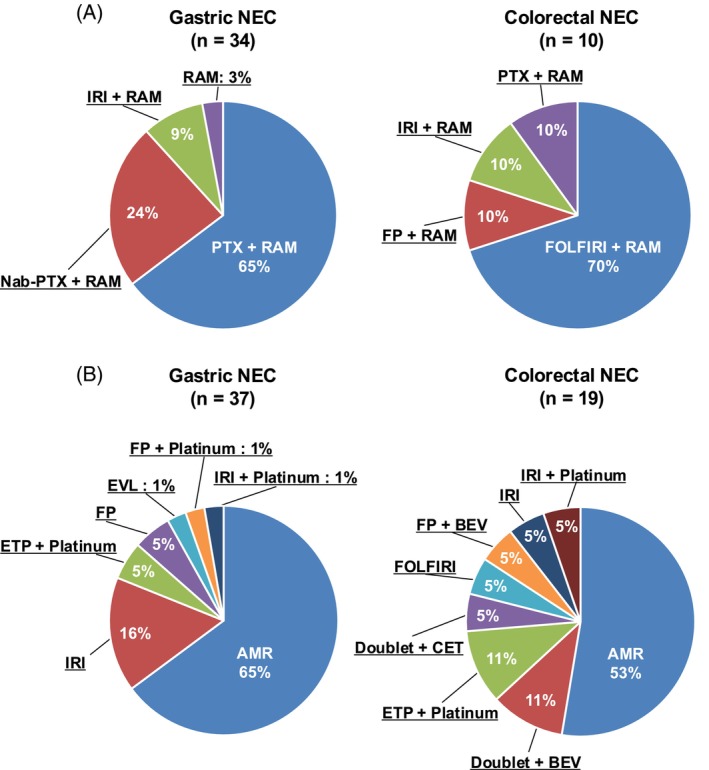
Treatment details. (A) Details of RAM‐containing chemotherapy for patients in the RAM group. (B) Details of second‐line treatments for patients in the non‐RAM group. AMR, amrubicin; BEV, bevacizumab; CET, cetuximab; Cx, chemotherapy; ETP, etoposide; EVL, everolimus; FP, fluorouracil; FTD/TPI, trifluridine/tipiracil; IRI, irinotecan; nabPTX, nab‐paclitaxel; NEC, neuroendocrine carcinoma; NGT, nogitecan; Nivo, nivolumab; Pembro, pembrolizumab; PTX, paclitaxel; RAM, ramucirumab; REGO, regorafenib.

Figure 1 shows the treatment details for both groups. The most commonly used RAM‐containing chemotherapy for patients with gastric NEC included paclitaxel plus RAM (*n* = 22, 65%), nab‐paclitaxel plus RAM (*n* = 8, 24%), and irinotecan plus RAM (*n* = 3, 9%), with one patient receiving RAM monotherapy (2%). Among patients with colorectal NEC, 70% (*n* = 7) received FOLFIRI plus RAM, and there was one patient each who received paclitaxel plus RAM, irinotecan plus RAM, and fluoropyrimidine plus RAM. In the RAM group, three patients with colorectal NEC had received FOLFOX plus bevacizumab as first‐line treatment. Six patients (17%) in the RAM group received amrubicin after RAM‐containing chemotherapy.

In contrast, 65% of patients with gastric NEC and 53% of patients with colorectal NEC were treated with amrubicin in the non‐RAM group. Other treatments involved platinum‐based chemotherapy in combinations different from those used in the first‐line treatment, irinotecan monotherapy, and fluoropyrimidine, as well as FOLFOX/FOLFIRI plus monoclonal antibodies such as bevacizumab, cetuximab, and panitumumab. In the non‐RAM group, no patients received VEGF inhibitors as first‐line treatment.

### 3.3 Efficacy

**FIGURE 2:**
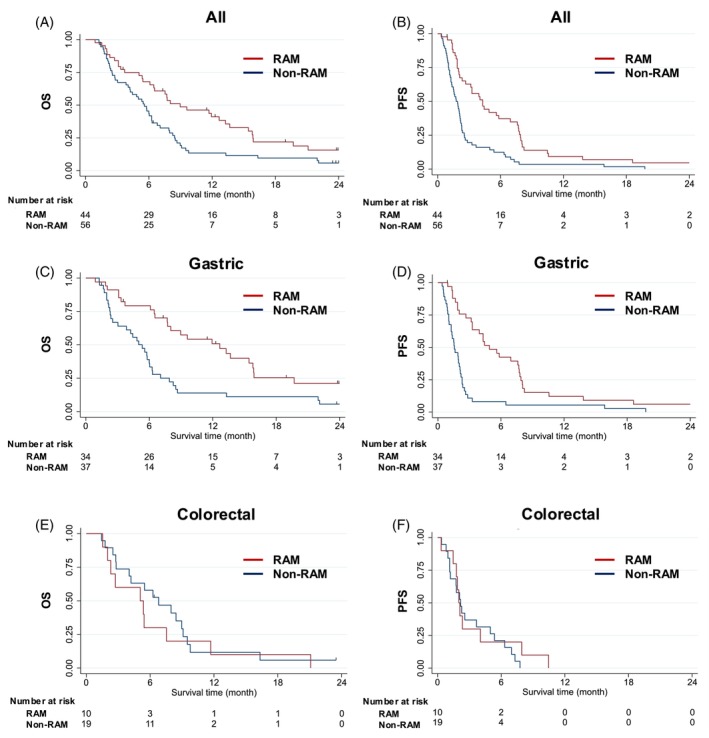
Kaplan–Meier curves of OS and PFS in patients diagnosed with NEC through central review. Kaplan–Meier survival curves comparing OS and PFS in patients with GI‐NEC treated with RAM and those not treated with RAM. (a) OS and (b) PFS for all patients. (c) OS and (d) PFS in patients with gastric NEC. (e) OS and (f) PFS in patients with colorectal NEC. GI‐NEC, gastrointestinal neuroendocrine carcinoma; OS, overall survival; PFS, progression‐free survival; RAM, ramucirumab.

The median OS was notably longer in the RAM group (9.0 months) than in the non‐RAM group (5.6 months), with a crude HR of 0.56 (95% CI: 0.36–0.87), an adjusted HR of 0.52 (95% CI: 0.26–1.02), and a PS‐IPW HR of 0.75 (95% CI: 0.45–1.28) (Figure 2 and Table 2). We observed a similar tendency in PFS, with a median PFS of 4.3 months in the RAM group and 1.8 months in the non‐RAM group, and a corresponding crude HR, an adjusted HR, and a PS‐IPW HR of 0.44 (95% CI: 0.29–0.67), 0.37 (95% CI: 0.20–0.66), and 0.45 (95% CI: 0.27–0.75), respectively. In patients with gastric NEC, we observed a median OS of 12.7 months in the RAM group compared with 5.3 months in the non‐RAM group, with a PS‐IPW HR of 0.56 (95% CI: 0.29–1.09). The median PFS was longer (4.9 months) in the RAM group than in the non‐RAM group (1.6 months), with a PS‐IPW HR of 0.32 (95% CI: 0.16–0.62). In patients with colorectal NEC, the distribution of the outcomes was small. The median OS was 5.1 months in the RAM group and 6.8 months in the non‐RAM group, with a PS‐IPW HR of 1.47 (95% CI: 0.52–4.18), whereas the median PFS was 2.0 months in the RAM group and 2.2 months in the non‐RAM group, with a PS‐IPW HR of 0.82 (95% CI: 0.26–2.61).

**TABLE 2:** OS and PFS in patients diagnosed with NEC through central review.

Population (RAM/Non‐RAM)	*N*	Median (months)	Crude HR (95% CI)	Adjusted HR (95% CI)	PS IPW HR (95% CI)
Overall survival					
All patients (*N* = 100)					
RAM group	44	9.0	0.56 (0.36–0.87)	0.52 (0.26–1.02)	0.75 (0.45–1.28)
Non‐RAM group	56	5.6			
Patients with gastric NEC (*N* = 71)					
RAM group	34	12.7	0.43 (0.25–0.73)	0.24 (0.11–0.54)	0.56 (0.29–1.09)
Non‐RAM group	37	5.3			
Patients with colorectal NEC (*N* = 29)					
RAM group	10	5.1	1.33 (0.60–2.94)	5.51 (0.93–32.8)	1.47 (0.52–4.18)
Non‐RAM group	19	6.8			
Progression‐free survival					
All patients					
RAM group	44	4.3	0.44 (0.29–0.67)	0.37 (0.20–0.66)	0.45 (0.27–0.75)
Non‐RAM group	56	1.8			
Patients with gastric NEC					
RAM group	23	4.9	0.35 (0.21–0.57)	0.24 (0.12–0.48)	0.32 (0.16–0.62)
Non‐RAM group	37	1.6			
Patients with colorectal NEC					
RAM group	7	2.0	0.74 (0.32–1.72)	1.85 (0.36–9.44)	0.82 (0.26–2.61)
Non‐RAM group	19	2.2			

Abbreviations: CI, confidence interval; HR, hazard ratio; IPW, inverse probability weighting; NEC, neuroendocrine carcinoma; RAM, ramucirumab.

A comparison limited to patients who received second‐line treatment showed that the RAM group had a better OS and PFS than the non‐RAM group, with this difference being particularly notable in gastric NEC (Table S3). Even when limiting the comparison to amrubicin as the second‐line treatment, a similar tendency was observed in the non‐RAM group (Table S4).

**FIGURE 3:**
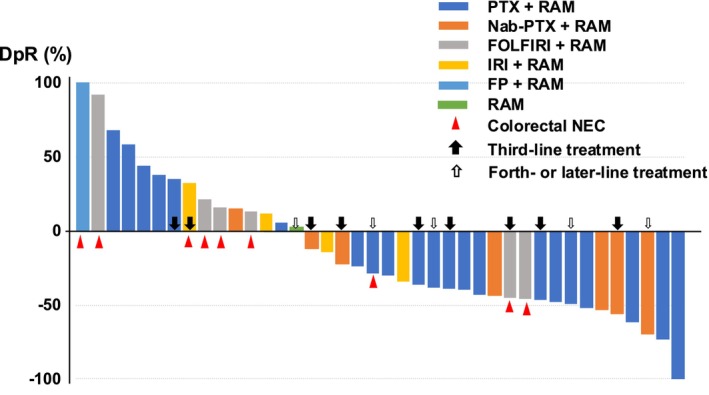
Waterfall plot for the RAM group in patients diagnosed with NEC through central review. The waterfall plot illustrates the depth of response percentage in patients with GI‐NEC undergoing RAM combined with chemotherapy. The median depth of response in the RAM group was −29.7%. GI‐NEC, gastrointestinal neuroendocrine carcinoma; RAM, ramucirumab.

Overall, the ORR in the RAM group was 44%, which was significantly higher than the 6% in the non‐RAM group (*p* < 0.001). A more detailed analysis of primary tumor location showed that in patients with gastric NEC, the RAM group had notably better ORR than the non‐RAM group (52% versus 3%, p < 0.001). However, for patients with colorectal NEC, the difference was less marked, with 20% in the RAM group and 11% in the non‐RAM group, indicating no significant difference (Table 3). The median depth of response in the RAM group was −29.7% (Figure 3). Notably, various treatments were utilized throughout chemotherapy, from the initial to subsequent treatments (Figure S5). Among the RAM‐containing chemotherapies, paclitaxel plus RAM was particularly effective, with an ORR of 62% (13/21). Conversely, there was no responder who received taxane alone, including paclitaxel and nab‐paclitaxel, as second‐ or later‐line treatments in the non‐RAM group (*n* = 5).

**TABLE 3:** Best response in patients diagnosed with NEC through central review.

Outcome	RAM group			Non‐RAM group			*p* value
	Overall	Gastric	Colorectal	Overall	Gastric	Colorectal	
	*N* = 41	*N* = 31	N = 10	*N* = 54	*N* = 35	*N* = 19	
ORR	44%	–	–	6%	–	–	<0.001
	–	52%	–	–	3%	–	<0.001
	–	–	20%	–	–	11%	0.59
DCR	64%	75%	30%	21%	11%	37%	–
Best response, *N* (%)							
CR	0	0	0	0	0	0	–
PR	18 (44)	16 (52)	2 (20)	3 (6)	1 (3)	2 (11)	–
SD	8 (20)	7 (23)	1 (10)	8 (15)	3 (8)	5 (26)	–
PD	14 (34)	8 (25)	6 (60)	41 (76)	29 (83)	12 (63)	–
NE	1 (2)	0	1 (10)	2 (3)	2 (6)	0	–

*Note*: Three patients in RAM group and two patients in non‐RAM group were excluded due to the absence of measurable lesions.

Abbreviations: CR, complete response; DCR, disease control rate; NE, not evaluable; ORR, objective response rate; PD, progressive disease; PR, partial response; RAM, ramucirumab; SD, stable disease.

### 3.4 Adverse events

In the RAM group, grade 3 and 4 adverse events were observed in 27% and 5% of the patients, respectively (Table S5). Neutropenia was the most frequent adverse event, occurring in 18% of patients at grade 3 and 2% at grade 4. One case of grade 4 febrile neutropenia was also found. One treatment‐related death due to rectal perforation occurred in a patient with colorectal NEC.

## 4 DISCUSSION

In this study, we assessed the clinical outcomes of RAM‐containing chemotherapy versus non‐RAM regimens in patients with refractory or platinum‐intolerant gastric or colorectal NEC. This study demonstrated that RAM‐containing chemotherapy as a second‐ or later‐line treatment may be effective for patients with GI‐NEC refractory to first‐line platinum‐based chemotherapy, with paclitaxel plus RAM showing remarkable efficacy in gastric NEC. To the best of our knowledge, this is the first study to evaluate the efficacy of RAM‐containing chemotherapy for GI‐NEC in a multicenter collaborative study involving a large number of patients (Table S6).

Angiogenesis has increasingly been recognized as a therapeutic target for various malignancies, including NEN. Several VEGFR tyrosine kinase inhibitors have been tested for advanced NENs, some of which have become standard treatments for NETs^23^, ^24^; however, evidence supporting the use of VEGF inhibitors for NECs is less robust than that for NETs. Previous reports suggested that vascular VEGFR‐2 expression in gastric NEC was higher than that reported on adenocarcinoma tissue in the REGARD trial. This might contribute to the remarkable tumor shrinkage in the present study.^20^, ^25^ The PRODIGE 41‐BEVANEC phase 2 trial evaluated FOLFIRI plus bevacizumab versus FOLFIRI alone in various GI‐NECs; however, it failed to demonstrate the benefit of adding the VEGF inhibitor bevacizumab.^12^ The primary tumor locations in the PRODIGE 41‐BEVANEC phase 2 trial were predominantly colorectum and pancreas, accounting for more than half of the study population. Notably, the prognosis for GEP‐NEC varies depending on the primary tumor location, and colorectal or pancreatic NEC has been observed to have worse prognoses than other forms. Analogously, the benefit of subsequent treatment, such as VEGF inhibitors, may differ by tumor location. The recent clinical trial for GEP‐NEC was conducted independent of the primary tumor location because of its rare frequency. However, the development of treatment stratified by primary tumor location may be needed to prolong the survival time for patients with GEP‐NEC. Our study suggests a limited benefit of RAM‐containing chemotherapy in colorectal NEC, potentially mirroring the findings of the PRODIGE 41‐BEVANEC trial, in which colorectal NEC was the most common subtype.

In the present study, RAM‐containing chemotherapy showed a tumor shrinkage of 43% among all patients and 52% in gastric NEC patients. Especially, paclitaxel plus RAM or nab‐paclitaxel plus RAM showed remarkable response rates. Disentangling whether the benefits of our study stem primarily from RAM or combination therapy is challenging. However, there were no responders who received taxane as a second‐ or later‐line treatment in the non‐RAM group. In addition, docetaxel showed an ORR of only 10.3% for extrapulmonary NEC in the NET‐02 study.^26^ These data may imply that the remarkable tumor shrinkage of RAM‐containing treatments in this study depends on the antitumor effect of RAM.

In our study, patients in the RAM group received RAM‐containing chemotherapy as second‐ or later‐line treatment. Notably, the RAM group included various treatment lines, while the non‐RAM group included only second‐line treatment. Thus, the RAM group potentially had a shorter survival period than the non‐RAM group. This implies that RAM‐containing chemotherapy prolongs OS and PFS; however, this tendency differs between patients with gastric NEC and those with colorectal NEC. In colorectal NEC, FOLFIRI plus ramucirumab is not necessarily superior, as other treatments with targeted therapy also demonstrate similar efficacy.

Regarding treatment details, the RAM and non‐RAM groups included various regimens. Patients with gastric NEC in the RAM group received RAM‐containing chemotherapies, which were the standard treatments for gastric adenocarcinoma. Paclitaxel plus RAM is a combination therapy established based on a phase 3 trial.^18^ In addition, phase 2 trials showed the efficacy and safety of nab‐paclitaxel plus RAM and irinotecan plus RAM for previously treated gastric adenocarcinoma.^27^, ^28^ Similarly, patients with colorectal NEC in the RAM group were treated with regimens such as FOLFIRI plus RAM, which was the standard second‐line treatment for colorectal adenocarcinoma. Only one patient with colorectal NEC received paclitaxel plus RAM. The majority of the RAM‐containing chemotherapy was paclitaxel plus RAM. Conversely, over half of the patients in the non‐RAM group received amrubicin, which is the recommended second‐line treatment for small‐cell lung cancer. Thus, the comparison between the RAM and non‐RAM group is actually a comparison between paclitaxel plus RAM and amrubicin. Amrubicin does not have many studies in GI‐NEC, mainly case series, and seems not used at all outside Japan.^4^, ^29^, ^30^, ^31^, ^32^, ^33^ When looking at the entire treatment line from first‐line to subsequent treatments, standard treatments for gastric adenocarcinoma, such as nivolumab or trifluridine/tipiracil (FTD/TPI), and colorectal cancer, such as FTD/TPI or regorafenib, were selected.^34^, ^35^ In patients centrally diagnosed as having gastric NEC, the difference of OS (12.7 vs. 5.3 months) was longer than that of PFS (4.9 vs. 1.6 months). The abundant subsequent treatments might contribute to this difference.

We conducted a central pathology review to increase the reliability of the findings in this study because distinguishing between NETs and NECs is sometimes difficult.^36^ Previous studies focusing on the effectiveness of second‐line treatment for GI‐NECs were performed in patients diagnosed according to the 2010 WHO classification; however, this classification included grade 3 NETs, which were re‐categorized as NETs according to the 2019 WHO classification. In NENs of the gastrointestinal tract, NETs have been shown to have different clinicopathological and molecular characteristics from those of NECs.^37^, ^38^ Therefore, we excluded 13 patients in whom Ki‐67 immunostaining was not evaluated because the possibility of NETs could not be excluded. Although the response of NETs to RAM is unknown, we believe that NETs should be excluded for the aim of this study.

This study has some limitations. First, it was a retrospective study; the non‐RAM group contained more frequent liver metastasis and fewer previous platinum responders. A higher proportion of previously treated patients in the RAM group suggests that these patients had a relatively slower disease progression, which allowed them to receive further chemotherapy. This imbalance between the two groups may have contributed to the difference in treatment outcomes. However, we aimed to reduce potential bias by adjusting the differences between the two groups using the IPW approach with the propensity score. Since the RAM group demonstrated favorable results in OS as well as PFS, the typical limitations of retrospective studies—such as non‐standardized CT scans and visit frequencies—likely had minimal impact on the findings. Second, this study combined patients with gastric and colorectal NECs. Therefore, when interpreting the overall results, the findings from organ‐specific subgroup analyses should be considered, as outcomes may differ between gastric and colorectal NECs. Third, potential indolent disease in patients who received more than two lines of previous treatment might have contributed to the survival advantage of the RAM group. However, even in analyses focusing on second‐line treatments, similar trends were observed as in the overall population, demonstrating reproducible results. In addition, this study contained both NEC and MANEC due to the difficulty of distinguishing between these entities from biopsy specimens. Although most patients were diagnosed as NEC, the present study could not show the outcomes of pure NEC. The strength of this study lies in the unusually large number of patients, especially considering NECs are rare; however, the subgroup analyses focused on the primary lesion and selected treatment, which contained a small number of patients.

This study suggested that RAM‐containing chemotherapy, especially paclitaxel plus RAM for gastric NEC, seems promising and should be further investigated.

## AUTHOR CONTRIBUTIONS

**Yuki Matsubara:** Conceptualization; methodology; data curation; investigation; formal analysis; funding acquisition; visualization; resources; writing – original draft; writing – review and editing. **Toshiki Masuishi:** Conceptualization; methodology; supervision; funding acquisition; resources; project administration; visualization; writing – original draft; writing – review and editing. **Waki Hosoda:** Resources; project administration; methodology; conceptualization; visualization; supervision; formal analysis; writing – original draft; writing – review and editing; investigation; validation. **Hidekazu Hirano:** Data curation; investigation; writing – review and editing. **Saori Mishima:** Data curation; investigation; writing – review and editing. **Hiroyuki Takahashi:** Data curation; investigation; writing – review and editing. **Tomoyuki Otsuka:** Data curation; investigation; writing – review and editing. **Kenta Kawasaki:** Data curation; investigation; writing – review and editing. **Takeshi Kawakami:** Data curation; investigation; writing – review and editing. **Kazuhiro Yanagihara:** Data curation; investigation; writing – review and editing. **Takaya Shimura:** Data curation; investigation; writing – review and editing. **Masato Komoda:** Data curation; investigation; writing – review and editing. **Kozue Murayama:** Data curation; investigation; writing – review and editing. **Keiko Minashi:** Data curation; investigation; writing – review and editing. **Yoshiyuki Yamamoto:** Data curation; investigation; writing – review and editing. **Yudai Shinohara:** Data curation; investigation; writing – review and editing. **Shinichi Nishina:** Data curation; investigation; writing – review and editing. **Nobuyuki Musha:** Data curation; investigation; writing – review and editing. **Kyoko Kato:** Data curation; investigation; writing – review and editing. **Kentaro Kawakami:** Data curation; investigation; writing – review and editing. **Katsunori Shinozaki:** Data curation; investigation; writing – review and editing. **Kenji Tsuchihashi:** Data curation; investigation; writing – review and editing. **Takayuki Ando:** Data curation; investigation; writing – review and editing. **Yosuke Kito:** Data curation; investigation; writing – review and editing. **Akitaka Makiyama:** Data curation; investigation; writing – review and editing. **Seiichiro Mitani:** Data curation; investigation; writing – review and editing. **Kaori Hino:** Data curation; investigation; writing – review and editing. **Naoki Izawa:** Data curation; investigation; writing – review and editing. **Isao Oze:** Conceptualization; methodology; software; resources; formal analysis; investigation; supervision; visualization; writing – original draft; writing – review and editing. **Kei Muro:** Conceptualization; methodology; supervision; funding acquisition; resources; project administration; writing – original draft; writing – review and editing.

## FUNDING INFORMATION

This investigator‐initiated work was supported by Eli Lilly Japan K.K., Japan. The funding bodies did not affect the study design, data collection, data analysis, interpretation, or writing of the manuscript.

## CONFLICT OF INTEREST STATEMENT

The authors declare the following conflicts of interest: Yuki Matsubara reports honoraria from Taiho, Chuagi, MSD, and Bristol‐Myers Squibb. Toshiki Masuishi reports honoraria from Bayer Yakuhin, Bristol‐Myers Squibb, Chugai Pharma, Daiichi‐Sankyo, Lilly, Merck Serono, Ono Pharmaceutical, Sanofi, Taiho Pharmaceutical, Takeda, Yakult Honsha, MSD, Kakata, Guardant Health, and Nippon Kayaku, and research funding from Amgen, Boehringer Ingelheim, CMIC, Daiichi‐Sankyo, Eli Lilly Japan, MSD, Novartis, Ono Pharmaceutical, Pfizer, and Syneos Health. Hidekazu Hirano reports honoraria from Novartis, Ono Pharmaceutical, Daiichi‐Sankyo, Taiho Pharmaceutical, and Bristol‐Myers Squibb and grants from PPD, Daiichi‐Sankyo, Nippon Boehringer Ingelheim, ALX Oncology, BeiGene, Novartis, Amgen, Bristol‐Myers Squibb, Seagen, and Taiho Pharmaceutical outside the submitted work. Saori Mishima reports honoraria from Taiho Pharmaceutical Co., Ltd., Chugai Pharmaceutical Co., Ltd., Takeda Pharmaceutical Co., Ltd., and Eli Lilly Co., Ltd. Hiroyuki Takahashi reports honoraria from AstraZeneca, Bristol‐Myers Squibb, Chugai Pharma, Janssen, Kyowa Kirin, Meiji Seika Pharma, Nippon Shinyaku, Mundipharma, Takeda Pharmaceutical, SymBio Pharmaceuticals, Ono Pharmaceutical, Eisai, and Sanofi S.A. Takeshi Kawakami is a review editor for *Frontiers in Oncology* and was not involved in the editorial review or the decision to publish this article and received honoraria from Eli Lilly. Masato Komoda reports honoraria from Lilly Japan, Ono Pharmaceutical, Bristol‐Myers Squibb Japan, Astellas, Daiichi Sankyo/UCB Japan, Taiho Pharmaceutical, Chugai Pharma, and MSD K.K. Takayuki Ando has received honoraria from Bristol‐Myers Squibb, Lilly Japan, Taiho Pharmaceutical, Daiichi Sankyo, Ono Pharmaceutical, Miyarisan Pharmaceutical, and Merck, and has received research funding from Chugai Pharma, Otsuka, Taiho Pharmaceutical, Daiichi Sankyo, and Nippon Kayaku. Akitaka Makiyama received honoraria from Eli Lilly Japan K.K., Taiho Pharmaceutical Co. Ltd., Ono Pharmaceutical Co. Ltd., Bristol‐Myers Squibb Co. Ltd., and Daiichi Sankyo Co. Ltd. Seiichiro Mitani received honoraria from Taiho Pharmaceutical Co., Ono Pharmaceutical Co., and MSD K.K., and participation on the advisory board of Chugai Pharmaceutical Co. Ltd. NI received grants from Taiho Pharmaceutical Co., honoraria from Taiho Pharmaceutical Co., Bristol‐Myers Squibb, Daiichi Sankyo, MSD, Sysmex, AstraZeneca, Nippon Kayaku, and Merck Serono. Kei Muro received honoraria from Ono, Takeda, Taiho, Bristol‐Myers Squibb, Eli Lilly, MSD, and Daiichi Sankyo; research funding from MSD, Amgen, ONO, Astellas, Sanofi, Taiho, PRA Health Sciences, PAREXEL International, Novartis, and Chugai; and consulting fees from AstraZeneca, Ono, Astellas, Chugai, and Amgen. Kei Muro was also a member of the advisory boards of AstraZeneca, Astellas, Amgen, and Takeda and an editorial board member of ESMO Gastrointestinal Oncology. Waki Hosoda, Tomoyuki Otsuka, Kenta Kawasaki, Kazuhiro Yanagihara, Takaya Shimura, Kozue Murayama, Keiko Minashi, Yoshiyuki Yamamoto, Yudai Shinohara, Shinichi Nishina, Nobuyuki Musha, Kyoko Kato, Kentaro Kawakami, Katsunori Shinozaki, Kenji Tsuchihashi, Yosuke Kito, Kaori Hino, and Isao Oze declare no conflict of interest.

## ETHICS STATEMENT

This study was approved by the Institutional Review Board of Aichi Cancer Center Hospital (R021156, UMIN000043200), and permission to conduct the study was obtained from the management of all participating facilities. All the experiments were performed in accordance with the principles of the Declaration of Helsinki. The Institutional Review Board approved the waiver of informed consent due to the observational retrospective study design, with an opportunity for patients to access study information and opt‐out provided on the institution's website.

## Supporting information


**DATA S1.** Supporting information.

## Data Availability

The data that support the findings of this study are available on request from the corresponding author.
