# Supplementary material for: Ramucirumab‐containing chemotherapy for patients with gastrointestinal neuroendocrine carcinoma refractory/intolerant to platinum‐based chemotherapy: A multicenter observational retrospective study (WJOG13420G)
Source: Int J Cancer. 2025 Jul 23;157(12):2577–88. doi: 10.1002/ijc.70053 (PMC12541556; doi:10.1002/ijc.70053)
Supplement: Supplementary file 1 — DATA S1. Supporting information. [file IJC-157-2577-s001.pdf]

Supplementary file

Ramucirumab-containing chemotherapy for patients with gastrointestinal neuroendocrine carcinoma refractory/intolerant to platinum-based chemotherapy: a multicenter observational retrospective study (WJOG13420G)

Yuki Matsubara, Toshiki Masuishi, Waki Hosoda, Hidekazu Hirano, Saori Mishima, Hiroyuki Takahashi, Tomoyuki Otsuka, Kenta Kawasaki, Takeshi Kawakami, Kazuhiro Yanagihara, Takaya Shimura, Masato Komoda, Kozue Murayama, Keiko Minashi, Yoshiyuki Yamamoto, Yudai Shinohara, Shinichi Nishina, Nobuyuki Musha, Kyoko Kato, Kentaro Kawakami, Katsunori Shinozaki, Kenji Tsuchihashi, Takayuki Ando, Yosuke Kito, Akitaka Makiyama, Seiichiro Mitani, Kaori Hino, Naoki Izawa, Isao Oze, Kei Muro

Table of Contents

Supplementary Figure S1. Study scheme

Supplementary Figure S2. Patient flow diagram

Supplementary Figure S3. Details of stratification

Supplementary Figure S4. Progression-free survival of first-line treatment

Supplementary Figure S5. Treatment sequence

Supplementary Table S1. Characteristics of patients by primary location

Supplementary Table S2. Efficacy of first-line treatment

Supplementary Table S3. Overall survival and progression-free survival of second-line treatment

Supplementary Table S4. Ramucirumab-containing chemotherapy vs. amrubicin in second-line treatment

Supplementary Table S5. Adverse events

Supplementary Table S6. Participating institutions

Supplementary Figure S1. Study scheme

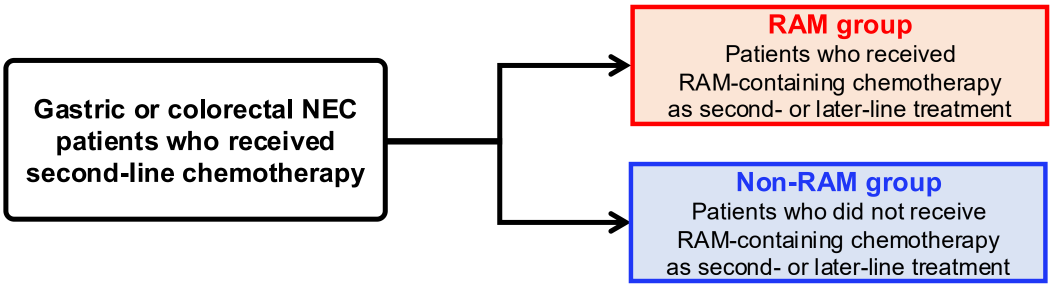

Patients were divided into two groups: patients who received RAM-containing chemotherapy as second-line treatment or beyond (the RAM group) and those who did not receive RAM-containing chemotherapy as any-line treatment (the non-RAM group).

RAM, ramucirumab

Supplementary Figure S2. Patient flow diagram

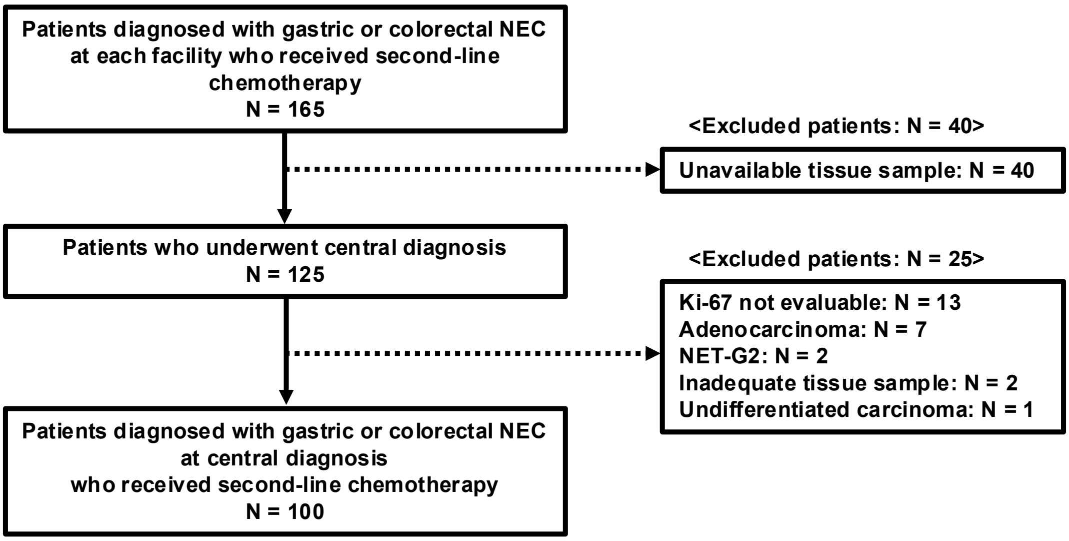

Second-line chemotherapy was administered to 165 patients with gastric or colorectal NEC diagnosed at each facility. The remaining 100 patients, validated as having NEC using the 2019 WHO classification, were the primary focus of the analysis.

NEC, neuroendocrine carcinoma; NET-G2, neuroendocrine tumor grade 2

Supplementary Figure S3. Details of stratification

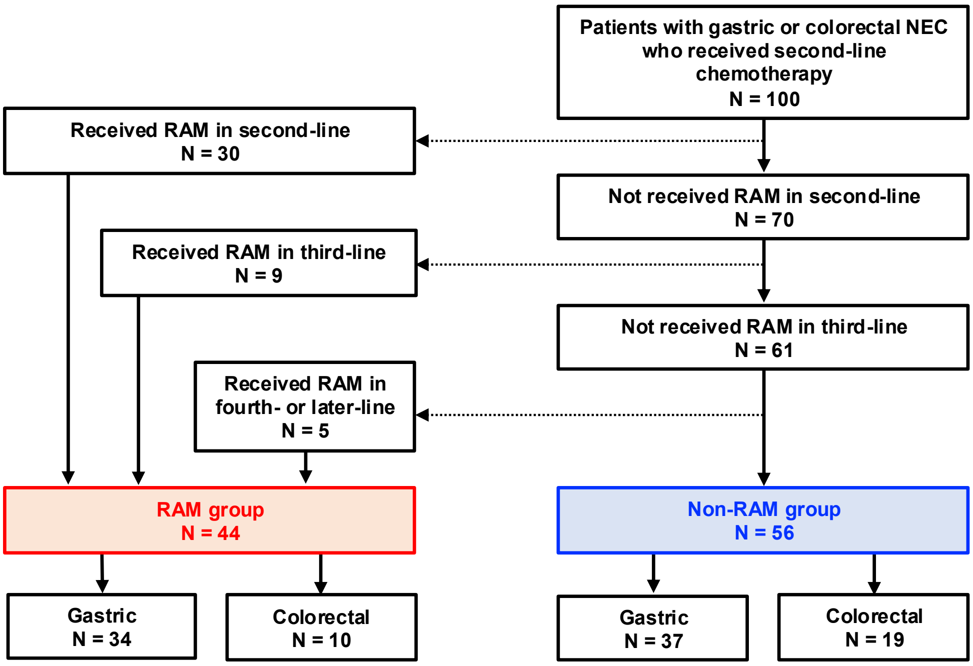

Stratification of patients with gastric or colorectal NEC who received second-line chemotherapy, detailing the distribution into the RAM and non-RAM groups.

NEC, neuroendocrine carcinoma; RAM, ramucirumab

Supplementary Figure S4. Progression-free survival of first-line treatment

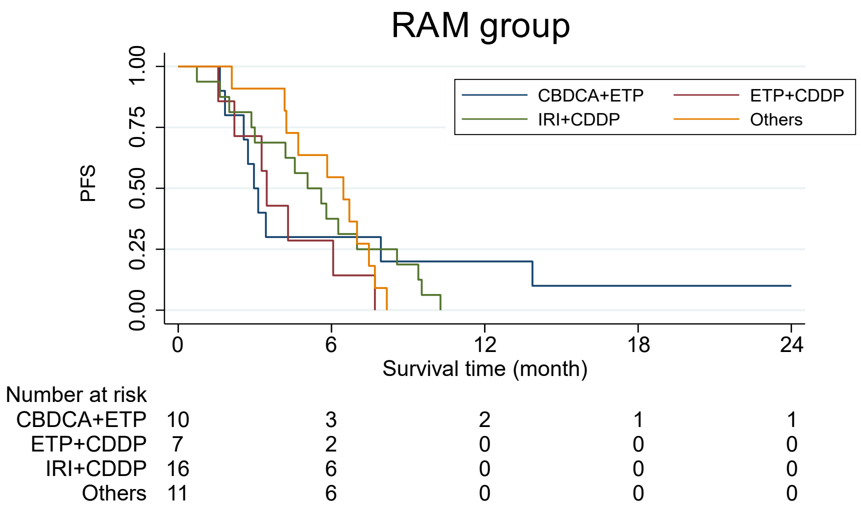

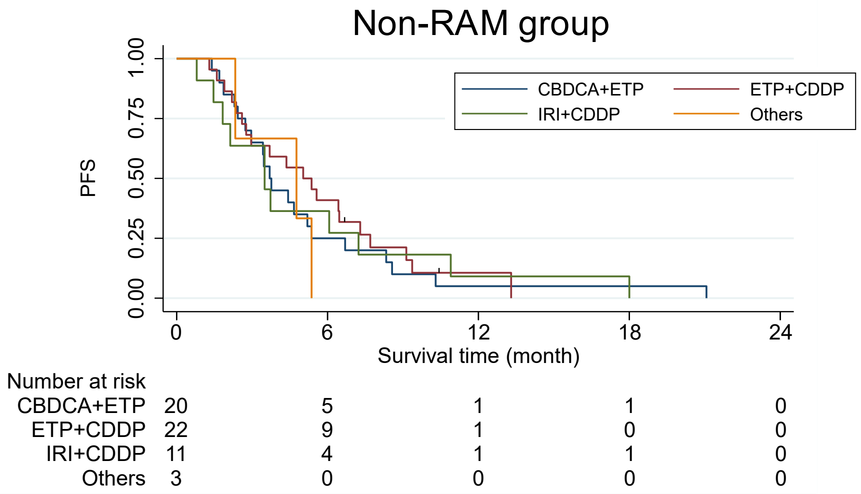

Kaplan–Meier survival curves of progression-free survival in first-line treatment.

RAM, ramucirumab; CBDCA, carboplatin; ETP, etoposide; CDDP, cisplatin; IRI, irinotecan

Supplementary Figure S5. Treatment sequence

(A) Treatment sequence in patients with gastric NEC

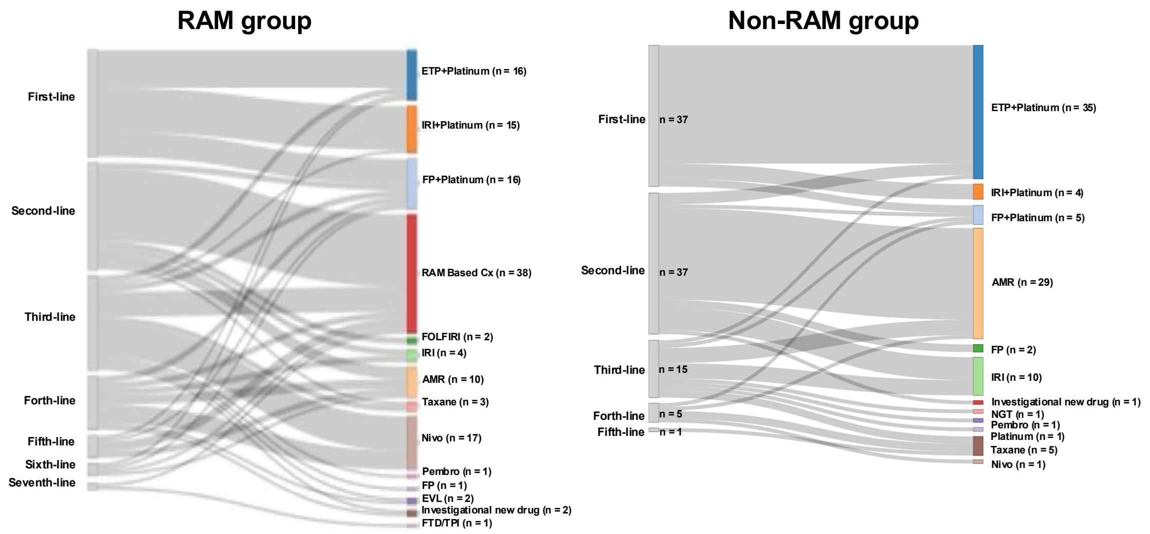

(B) Treatment sequence in patients with colorectal NEC

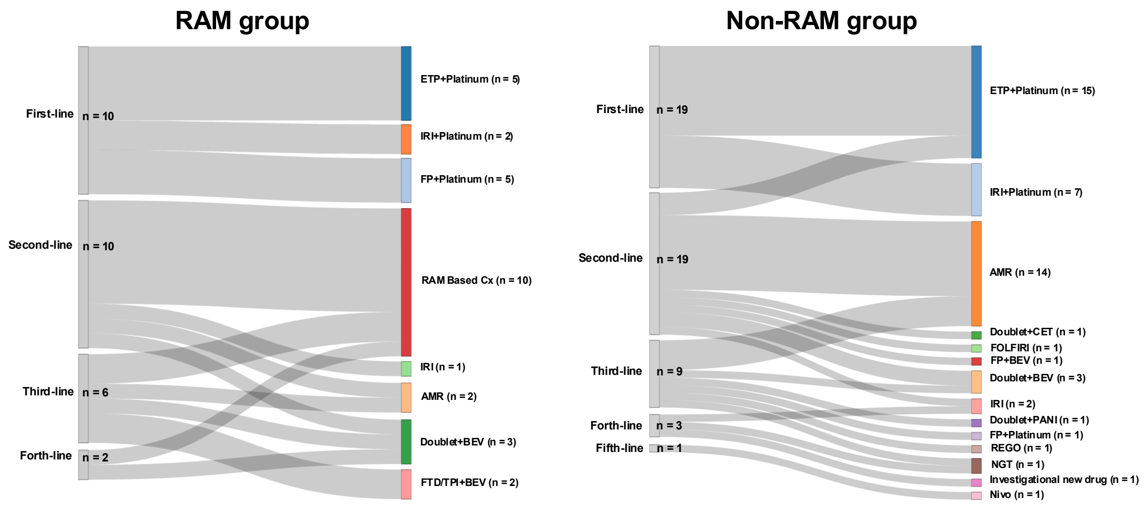

The Sankey diagram illustrates the treatment sequences for patients in the RAM and non-RAM groups. Each horizontal band represents a line of treatment from first- to seventh-line therapies.

AMR, amrubicin; BEV, bevacizumab; CET, cetuximab; Cx, chemotherapy; ETP, etoposide; EVL, everolimus; FP, fluorouracil; FTD/TPI, trifluridine/tipiracil; IRI, irinotecan; nabPTX, nab-paclitaxel; NEC,

neuroendocrine carcinoma; NGT, nogitecan; Nivo, nivolumab; Pembro, pembrolizumab; PTX, paclitaxel; RAM, ramucirumab; REGO, regorafenib

Supplementary Table S1. Characteristics of patients by primary location

**Factor**		**RAM group**		**Non-RAM group**	
		Gastric NEC<br>N = 34	Colorectal NEC<br>N = 10	Gastric NEC<br>N = 37	Colorectal NEC<br>N = 19
**Age (years)**	Median (range)	70<br>(54–83)	64<br>(46–80)	70<br>(41–81)	66<br>(43–81)
**Sex**	Male	30 (88%)	6 (60%)	32 (86%)	9 (47%)
	Female	4 (12%)	4 (40%)	5 (14%)	10 (53%)
**Histology**	NEC	28 (82%)	8 (80%)	35 (95%)	17 (89%)
	MANEC	6 (18%)	2 (20%)	2 (5%)	2 (11%)
**Time of first metastasis**	Synchronous	21 (62%)	7 (70%)	32 (86%)	14 (74%)
	Metachronous	13 (38%)	3 (30%)	5 (14%)	5 (26%)
**Ki-67**	Median (range)	80<br>(30–100)	90<br>(50–90)	80<br>(70–100)	80<br>(40–100)
	≥ 50%	31 (91%)	10 (100%)	37 (100%)	18 (95%)

	< 50%	3 (9%)	0	0	1 (5%)
**ECOG PS**	0	15 (44%)	5 (50%)	15 (41%)	7 (37%)
	1	17 (50%)	4 (40%)	18 (49%)	11 (58%)
	2	2 (6%)	1 (10%)	4 (11%)	1 (5%)
**Number of prior regimens**	1	23 (68%)	7 (70%)	37 (100%)	19 (100%)
	2	7 (21%)	2 (20%)	0	0
	≥ 3	4 (12%)	1 (10%)	0	0
**Response to platinum**	Yes	17 (50%)	6 (60%)	10 (27%)	4 (21%)
	No	17 (50%)	4 (40%)	27 (73%)	15 (79%)
**Previous primary resection**	Yes	13 (38%)	5 (50%)	7 (19%)	9 (47%)
	No	21 (62%)	5 (50%)	30 (81%)	10 (53%)
**Number of metastatic lesions**	1	16 (47%)	4 (40%)	14 (38%)	4 (21%)
	≥ 2	18 (53%)	6 (60%)	23 (62%)	15 (79%)
**Liver metastasis**	Yes	25 (74%)	8 (80%)	35 (95%)	16 (84%)

	No	9 (26%)	2 (20%)	2 (5%)	3 (16%)
**Lung metastasis**	Yes	2 (6%)	0	4 (11%)	7 (37%)
	No	32 (94%)	10 (100%)	33 (89%)	12 (63%)
**Peritoneal metastasis**	Yes	8 (24%)	2 (20%)	3 (8%)	8 (42%)
	No	25 (76%)	8 (80%)	34 (92%)	11 (58%)

Supplementary Table S2. Efficacy of first-line treatment

Treatment	Group	N	ORR (%)	DCR (%)	Median PFS (months)
Carboplatin plus etoposide	RAM	10	50.0	62.5	3.9
	Non-RAM	20	15.6	63.2	3.7
Cisplatin plus etoposide	RAM	7	71.4	71.4	3.5
	Non-RAM	22	30.0	65.0	5.0
Carboplatin plus etoposide	RAM	16	43.8	81.3	5.1
	Non-RAM	11	27.3	54.5	3.5
Others	RAM	11	70.0	90.0	6.5
	Non-RAM	3	50.0	50.0	4.8

Supplementary Table S3. Overall survival and progression-free survival of second-line treatment

Population (RAM/Non-RAM)	N	Median (months)	Crude HR (95% CI)	Adjusted HR (95% CI)	PS IPW HR (95% CI)
**Overall survival**					
**All patients**					
**RAM group**	30	7.7	0.61 (0.37–0.99)	0.56 (0.29–1.09)	0.76 (0.45–1.28)
**Non-RAM group**	56	5.6			
**Patients with gastric NEC**					
**RAM group**	23	11.9	0.46 (0.25–0.82)	0.27 (0.12–0.60)	0.56 (0.29–1.09)
**Non-RAM group**	37	5.3			
**Patients with colorectal NEC**					
**RAM group**	7	2.7	1.95 (0.79–4.82)	4.69 (0.84–26.3)	1.48 (0.52–4.18)
**Non-RAM group**	19	6.8			
**Progression-free survival**					
**All patients**					
**RAM group**	30	3.3	0.48 (0.30–0.76)	0.42 (0.23–0.76)	0.45 (0.27–0.75)

**Non-RAM group**	56	1.8			
**Patients with gastric NEC**					
**RAM group**	23	4.3	0.36 (0.20–0.63)	0.27 (0.13–0.57)	0.32 (0.16–0.61)
**Non-RAM group**	37	1.6			
**Patients with colorectal NEC**					
**RAM group**	7	1.9	1.03 (0.40–2.64)	1.60 (0.32–8.02)	0.82 (0.26–2.61)
**Non-RAM group**	19	2.2			

CI, confidence interval; HR, hazard ratio; IPW, inverse probability weighting; NEC, neuroendocrine carcinoma; RAM, ramucirumab

Supplementary Table S4. Ramucirumab-containing chemotherapy vs. amrubicin in second-line treatment

Population (RAM/Non-RAM)	N	Median (months)	Crude HR (95% CI)	Adjusted HR (95% CI)	PS IPW HR (95% CI)
**Overall survival**					
**All patients**					
**RAM group**	30	7.7	0.67 (0.39–1.14)	0.68 (0.32–1.42)	0.82 (0.46–1.44)
**Non-RAM group**	34	5.8			
**Patients with gastric NEC**					
**RAM group**	23	11.9	0.56 (0.30–1.05)	0.32 (0.13–0.81)	0.68 (0.34–1.37)
**Non-RAM group**	24	5.6			
**Patients with colorectal NEC**					
**RAM group**	7	2.7	1.41 (0.49–4.05)	NE	1.05 (0.34–3.25)
**Non-RAM group**	10	6.8			
**Progression-free survival**					
**All patients**					
**RAM group**	30	3.3	0.50 (0.33–0.76)	0.45 (0.27–0.73)	0.58 (0.39–0.88)

**Non-RAM group**	34	1.9			
**Patients with gastric NEC**					
**RAM group**	23	4.3	0.42 (0.23–0.77)	0.31 (0.13–0.74)	0.41 (0.19–0.86)
**Non-RAM group**	24	1.6			
**Patients with colorectal NEC**					
**RAM group**	7	1.9	1.13 (0.40–3.18)	NE	0.85 (0.28–2.60)
**Non-RAM group**	10	2.1			

CI, confidence interval; HR, hazard ratio; IPW, inverse probability weighting; NEC, neuroendocrine carcinoma; RAM, ramucirumab

Supplementary Table S5. Adverse events

Adverse events in patients diagnosed with NEC through central review

Adverse events (N = 44)	Grade 3	Grade 4
**Any**	12 (27%)	2 (5%)
**Neutropenia**	8 (18%)	1 (2%)
**Febrile neutropenia**	0	1 (2%)
**Anemia**	1 (2%)	0
**Leukopenia**	1 (2%)	0
**Skin pigmentation**	0	0
**Anorexia**	2 (5%)	0
**Fatigue**	0	0
**Fever**	1 (2%)	0
**Infection**	1 (2%)	0
**Anal fistula**	1 (2%)	0

One treatment-related death occurred because of rectal perforation.

Supplementary Table S6. Participating institutions

Institution	Representative Investigator
**Keiyukai Sapporo Hospital**	Kentaro Kawakami
**Faculty of Medicine, University of Tsukuba**	Yoshiyuki Yamamoto
**Saitama Cancer Center**	Kozue Murayama
**Chiba Cancer Center**	Keiko Minashi
**National Cancer Center Hospital East**	Saori Mishima
**National Cancer Center Hospital**	Hidekazu Hirano
**Keio University School of Medicine**	Kenta Kawasaki
**St. Marianna University School of Medicine Hospital**	Naoki Izawa
**Kanagawa Cancer Center**	Hiroyuki Takahashi
**Saiseikai Niigata Hospital**	Nobuyuki Musya
**Shizuoka Cancer Center Hospital**	Takeshi Kawakami
**Gifu University Hospital**	Akitaka Makiyama
**Toyama University Hospital**	Takayuki Ando
**Ishikawa Prefectural Central Hospital**	Yosuke Kito
**Aichi Cancer Center Hospital**	Yuki Matsubara
**Nagoya City University Graduate School of Medical Sciences**	Takaya Shimura
**National Hospital Organization Nagoya Medical Center**	Kyoko Kato
**Osaka International Cancer Institute**	Tomoyuki Otsuka
**Kansai Electric Power Hospital**	Kazuhiro Yanagihara
**Faculty of Medicine, Kindai University Hospital**	Seiichiro Mitani
**Kurashiki Central Hospital**	Shinichi Nishina

**Hiroshima Prefectural Hospital**	Katsunori Shinozaki
**NHO Shikoku Cancer Center**	Kaori Hino
**Japan Community Healthcare Organization Kyushu Hospital**	Yudai Shinohara
**NHO Kyushu Cancer Center**	Masato Komoda
**Kyushu University Hospital**	Kenji Tsuchihashi
